# Cytoprotective Effects of Mangiferin and *Z*-Ligustilide in PAH-Exposed Human Airway Epithelium in Vitro

**DOI:** 10.3390/nu11020218

**Published:** 2019-01-22

**Authors:** Dovilė Grauzdytė, Jovilė Raudoniūtė, Ieva Kulvinskienė, Edvardas Bagdonas, Inga Stasiulaitienė, Dainius Martuzevičius, Daiva Bironaitė, Rūta Aldonytė, Petras Rimantas Venskutonis

**Affiliations:** 1 Department of Food Science and Technology, Kaunas University of Technology, Radvilėnų pl. 19, Kaunas LT-50254, Lithuania; dovile.grauzdyte@ktu.lt; 2 State research Institute Center for Innovative Medicine, Santariskiu 5, Vilnius 08406, Lithuania; jovile.raudoniute@imcentras.lt (J.R.); ieva.bruzauskaite@imcentras.lt (I.K.); Edvardas.bagdonas@imcentras.lt (E.B.); daiva.bironaite@imcentras.lt (D.B.); ruta.aldonyte@imcentras.lt (R.A.); 3 Department of Environmental Technology, Kaunas University of Technology, Radvilėnų pl. 19, Kaunas LT-50264, Lithuania; Inga.stasiulaitiene@ktu.lt (I.S.); Dainius.martuzevicius@ktu.lt (D.M.)

**Keywords:** PAH, mangiferin, *Z*-ligustilide, BEAS-2B, cytotoxicity, antioxidant capacity

## Abstract

According to World Health Organisation (WHO) air pollution increases the risk of cardiovascular disorders, respiratory diseases, including COPD, lung cancer and acute respiratory infections, neuro-degenerative and other diseases. It is also known that various phytochemicals may mitigate such risks. This study tested if phytochemicals mangiferin (MNG) and Z-ligustilide (Z-LG) may protect PAH-exposed human lung bronchial epithelial cells (BEAS-2B). Organic PAH extract was obtained from the urban fine PM with high benzo(a)pyrene content collected in Eastern European mid-sized city during winter heating season. Cell proliferation traits and levels of intracellular oxidative stress were examined. Effect of MNG (0.5 µg/mL) alone or in combination with PAH on bronchial epithelium wound healing was evaluated. Both phytochemicals were also evaluated for their antioxidant properties in acellular system. Treatment with MNG produced strong cytoprotective effect on PAH-exposed cells (*p* < 0.01) while *Z*-LG (0.5 µg/mL) exhibited strong negative effect on cell proliferation in untreated and PAH-exposed cells (*p* < 0.001). MNG, being many times stronger antioxidant than *Z*-LG in chemical in vitro assays (*p* < 0.0001), was also able to decrease PAH-induced oxidative stress in the cell cultures (*p* < 0.05). Consequently MNG ameliorates oxidative stress, speeds up wound healing process and restores proliferation rate in PAH-exposed bronchial epithelium. Such protective effects of MNG in air pollution affected airway epithelium stimulate further research on this promising phytochemical.

## 1. Introduction

Mitigation of the air pollution-linked adverse health effects is among the highest priorities of modern society in parallel with the efforts to reduce air pollution. A number of traditional epidemiological studies have demonstrated a relationship between the exposure to ambient particulate matter (PM) and development of cancer, cardiovascular and respiratory illnesses [1,2]. PM is a complex mixture of organic and inorganic components, usually defined as PM10, PM2.5 and PM1, corresponding to airborne particles with an aerodynamic diameter equal or less than 10, 2.5 and 1 micron, respectively. PM10 and PM2.5 are often classified as the “coarse” fractions; while ≤PM2.5 as the “fine” one [3]. Polycyclic aromatic hydrocarbons (PAH) with two or more fused benzene rings are formed during the incomplete combustion of organic materials such as coal, oil, petrol, wood and due to their ubiquitous occurrence in urban air PM, bioaccumulation potential and carcinogenic activity, present significant environmental concern.

Air pollution-exposed organisms and cells exhibit increased levels of oxidative stress, including mitochondrial dysfunction and augmented production of reactive oxygen species (ROS) [4]. Data from large clinical trials further support an association of air pollution with circulating biomarkers of oxidative stress in humans [5]. Growing body of evidence suggests that certain antioxidants may mitigate the risks of increased oxidative stress and subsequent development of pollution associated diseases, like cancer, COPD, cardiovascular disorders, cataract, age-related macular degeneration and other aging-related diseases [6]. Most of the human diseases associated with increased air pollution are defined by the irreversible chronic inflammation, which affects airways rendering them narrowed and obstructed and plays a crucial role in destruction of respiratory tissue, that is, alveoli [7]. Chronic inflammation is followed by modification of structural proteins turning them into auto-antigens and excessive generation of ROS among other factors [8]. Moreover, PAH contribute to tumorigenesis, secretion of inflammatory cytokines, other pro-inflammatory changes, hypersensitivity reactions and induced auto immunity [9,10]. To date, several studies have confirmed that some harmful effects of air pollution may be modified by the intake of fruits, vegetables, antioxidant vitamins such as C, E, β-carotene and polyunsaturated fatty acids [11,12,13].

The growing interest in finding new protective phytochemicals and evaluation of their bioactive properties encourages the search for new natural compounds, which could mitigate the adverse health effects associated with air pollution. In this respect, mangiferin (a natural bioactive xanthone C-glycoside, further abbreviated as MNG) and *Z*-ligustilide (a phthalide derivative, further abbreviated as *Z*-LG), which are known for various beneficial health properties, are attractive potential candidates. MNG is produced by a variety of plant families such as Mangiferaceae, Zingiberaceae, Celastraceae, Gentianaceae and Aphloiaceae [14]. For instance, the peels of mango fruits were recently found to be a particularly rich source of MNG [15]. For centuries in the traditional healing practices in India and China the use of plants containing MNG was an important tool in disease management and health benefits. A number of pharmacological studies has proved its effectiveness as an antioxidant [16], analgesic, anti-inflammatory [17], antidiabetic [18], cardioprotective [19], photoprotective [20] and anticancer [21] agent. *Z*-LG is found in some medicinal plants including Cnidii Rhizoma, *Angelica sinensis* and *Levisticum officinale* and is also widely recognized as an anti-inflammatory agent and anti-oxidant [22,23,24]. Recently, the pharmacological activity of *Z*-LG has been investigated and it has been shown to exhibit neuroprotective [25], anti-Alzheimer [22], anticancer [26] and anti-inflammatory [27] effects. It is important to note that bioavailability, that is defined as substance’s presence in the circulatory system and target tissues, may be a limiting factor in preventive and other beneficial functions of these compounds. However, significant dietary intake-associated increase in MNG blood level was reported [28] and suggests that most body tissues, including bronchial epithelium, may benefit from the dietary intake of this phytochemical.

To our knowledge, the protective effects of MNG and *Z*-LG on PAH-damaged bronchial epithelium were not studied previously. Thus, the major objective of the present study was to investigate probable protective effects of MNG and *Z*-LG on the viability and redox state of PAH-exposed human bronchial epithelial (BEAS-2B) cells. MNG effect on cells motility was also investigated.

## 2. Materials and Methods

### 2.1. Cell Cultures

Human bronchial epithelial cells BEAS-2B, SV40-adenovirus-hybrid (Ad12SV40) were obtained from Sigma-Aldrich (Steinheim, Germany). Cell culture was maintained in F-12 K medium (Thermo Scientific, Waltham, MA, USA) supplemented with 10% FBS and 1% antibiotics (Biochrom, Berlin, Germany). The medium was changed twice weekly. BEAS-2B cells were maintained in serum-free medium for 12 h prior to and during the experiments.

### 2.2. Chemicals and Reagents

Diphenyl-1-picryhydrazyl stable radical (DPPH^•^, 98%), 2,2-azino-bis(3-ethylbenzothiazoline-6- sulfonic acid) diammonium salt (ABTS, 98%), fluorescein (FL), 2,2-azobis(2-amidinopropane) dihydrochloride AAPH, 6-hydroxy-2-5-7-8-tetramethylchroman-2-carboxylic acid (Trolox, 97%), 2,4,6-tripyridyl-s-triazine (TPTZ), mangiferin and dimethyl sulfoxide (DMSO) were purchased from Sigma-Aldrich (Steinheim, Germany). KCl, Na_2_HPO_4_, K_2_S_2_O_8_ and NaCl were purchased from Merck (Darmstadt, Germany). KH_2_PO_4_ was from Jansen Chimica (Beerse, Belgium). Methanol and acetic acid were obtained from StanLab (Lublin, Poland). Z-LG was purchased from Chengdu Biopurify Phytochemicals (China).

### 2.3. Sampling, Analysis and Preparation of PAH Extract

The PM1 samples were collected in the city of Kaunas, Lithuania, with ca 300 thousand inhabitants. The sampling was performed in busy part of the city during 12–25 December 2016.

The fine fraction of urban aerosol represented by PM1 was collected using the high volume aerosol sampler (Digitel AG, DH-77) and PAH extracted and analysed as previously described [29]. The PM1 cut-off has been selected since the major part of the PM mass in winter period consists of sub-micrometre particles (PM1), which are the product of solid fuel combustion processes in decentralized heating boilers (30). The sub-micrometre particle fraction has been also recognized for its high toxicity, resulting from its small size, high surface area and capability in adsorbing multiple pollutant species (hydrocarbons, heavy metals, ions etc.).

PM loaded filters were cut, submerged in DMSO and sonicated in ultrasonic water bath 3 times for 10 min. Resulting solution was filtered (0.2 µm pore size) and stored at −20 °C. PAH content was analysed and extract was added directly to the cell culture medium. Its working dilution was calculated based on environmental level of benzo(a)pyrene (BaP) in the Eastern European city (Kaunas, Lithuania) centre during heating season, that is, 3 ng/m^3^ as detected in our study. An estimated level of BaP in the bronchial lining fluid of a person exposed to 3 ng/m^3^ of ambient BaP might be approximately 2 nM based on other studies [30]. Working concentration of PAH extract was adjusted accordingly and constituted 20 μL/mL. Control cells were treated with solvent, that is, DMSO, only. Treatment was applied for 24, 48 and 72 h as indicated below. Both phytochemical compounds were added at the level of 0.5 µg/mL.

### 2.4. Cytotoxicity and Wound Healing Assays

BEAS-2B cells were grown in a 48-well plate at density of 15,000 cells/well. Cells were kept in serum free F-12 medium for 12 h prior to the exposure to PAH extract (containing 2 nM of BaP) with or without phytochemicals, that is, 0.5 µg/mL of Z-LG and MNG. Cells were treated for 24, 48 and 72 h and proliferation was determined by using CCK-8 kit according to manufacturer’s instructions (Dojindo Molecular Technologies, Rockville, MD, USA). This tetrazolium salt-based assay provides reliable and convenient platform to test toxicity. Tetrazolium salt WST-8 is reduced by cellular dehydrogenases to water soluble orange coloured product (formazan) and the intensity of the colour is determined spectrophotometrically.

Scratch assay was carried out to determine the wound healing abilities of BEAS-2B cells after the exposure to PAH extract and phytochemicals. BEAS-2B cells were seeded into 6-well plates and grown until 80–90% confluence. The cell monolayer was scratched with a sterile 1 mL pipette tip. Subsequent imaging at the beginning and at the regular intervals during the coverage of the wounded area followed. Images are represented to visually rate the closure of the gap in exposed and control cultures.

### 2.5. Detection of ROS

Cells were seeded into glass-bottom plates, grown to confluence and exposed to PAH extract (containing 2 nM of BaP), *Z*-LG (0.5 µg/mL), MNG (0.5 µg/mL) and combinations of them for 24 h. Next, CellROX Green detection reagent (Molecular Probes, Life technologies) was added to the test wells according to manufacturer’s suggestions (at the final concentration of 5 µM). CellROX^®^ Green Reagent is a fluorogenic probe designed to measure oxidative stress in the live cells. This cell-permeant dye is weakly fluorescent at the normal conditions, while in a reduced state exhibits bright green fluorescence corresponding to the amount of ROS within the cells and subsequent their binding to DNA with absorption/emission maxima at 485/520 nm. After incubation for 1 h at 37 °C the cells were washed and analysed under fluorescent Nikon Eclipse TE 2000-U microscope (Nikon Instruments, Tokyo, Japan).

Quantitative assessment of the oxidative stress in the cultures was also performed. Muse^®^ Oxidative Stress Kit (Millipore Sigma) and Muse^®^ cytometer were used according to manufacturer’s instructions. Relative percentage of ROS negative and ROS positive cells was counted by the Muse^®^ Cell Analyzer. Kit utilizes dihydroethidium-based reagent that has been extensively used to detect ROS, namely superoxide radicals, in cell populations and is compatible with Muse™ Cell Analyzer, where intuitive software provides detailed analysis of cells distinguishing between two populations, that is, ROS(−) and ROS(+) cells.

### 2.6. In Vitro Antioxidant Activity Assessment

The antioxidant activity of the extracts was determined by different antioxidant assays mentioned below. For the measurements, MNG and *Z*-LG were dissolved in methanol at a concentration of 10 mg/mL and further diluted to a final concentration (0.001–0.25%), suitable for selected assay. The absorbance was measured with Spectronic Genesys 8 spectrophotometer (Thermo Spectronic, Rochester, NY, USA) in semi-micro cuvettes (Ratiolab GmbH, Dreieich, Germany).

#### 2.6.1. DPPH^•^ Scavenging Assay

The DPPH^•^ scavenging assay was carried out by the method of Brand-Williams et al. [31] with some modifications. For the analysis 1000 µL of DPPH^•^ methanolic solution (~90 µmol/L, final absorption 0.800 ± 0.03) was mixed with 500 µL of tested phytochemicals solutions or MeOH (blank) and left in dark for 2 h. A series of Trolox solutions (0–50 µmol TE/L MeOH) were used for calibration. The decrease in absorbance value was measured at 517 nm. Radical scavenging capacity was calculated by the formula:

% inhibition = ((AB − AE)/AB) × 100
(1)

where AB—absorption of blank sample; AE—absorption of the sample with MNG or Z-LG. The results were expressed in µM of Trolox equivalents per g of tested phytochemical (µM TE/g).

#### 2.6.2. ABTS^•+^ Scavenging Assay

The ABTS^•+^ scavenging assay was carried out by the method of Re et al. [32], with slight modifications. Firstly, phosphate buffered saline (PBS) solution (75 mM/L; pH 7.4) was prepared by dissolving 8.18 g NaCl, 0.27 g KH_2_PO_4_, 3.58 g Na_2_HPO_4_ × 12 H_2_O and 0.15 g KCl in 1 L of distilled water. Stock ABTS^•+^ solution was prepared by mixing 50 mL ABTS (2 mM/L PBS) with 200 µL K_2_S_2_O_8_ (70 mM/L H_2_O) and keeping for 12–16 h at room temperature in the dark. Before each assay, stock ABTS^•+^ solution was diluted with PBS to obtain the working ABTS^•+^ solution with absorbance of 0.80 ± 0.03 at 734 nm. For the analysis, 25 µL of tested phytochemicals solutions or MeOH (blank) were mixed with 1500 µL of working ABTS^•+^ solution and left in dark for 2 h. A series of Trolox solutions in the concentration ranges of 0–1500 µmol/L MeOH were used for the calibration. Radical scavenging capacity was calculated as in the DPPH^•^ assay and the results were expressed in µM TE/g.

#### 2.6.3. Ferric Reducing Antioxidant Power (FRAP) Assay

FRAP assay was carried out using the method of Benzie and Strain [33] with some modifications. FRAP reagent was prepared by mixing a solution of 10 mM TPTZ (in 40 mM HCl), 20 mM FeCl_3_∙6H_2_O and acetate buffer (300 mM, pH 3.6) at 1:1:10 (*v*/*v*/*v*). For the measurement, 50 µL of sample or MeOH (blank) were mixed with 150 µL of distilled H_2_O and 1500 µL of freshly prepared FRAP reagent. After 2 h incubation in the dark, the decrease in absorbance was read at 593 nm. A series of Trolox solutions in the concentration ranges of 0–800 µmol/L MeOH were used for the calibration and the results were expressed in µM TE/g.

#### 2.6.4. Oxygen Radical Absorbance Capacity (ORAC) Assay

ORAC assay was carried out by using fluorescein as a fluorescent probe [34]. For the analysis 25 µL of sample or MeOH (blank) were mixed with 150 µL of fluorescein solution (14 µmol/L PBS) in a black, clear-bottom, 96-well black opaque microplate. The mixture was preincubated for 15 min at 37 °C and 25 µL of AAPH solution (240 mmol/L PBS) as a peroxyl radical generator immediately added using multichannel pipet. The fluorescence was recorded every 1 min at 485 excitation and 520 emission wavelengths during 120 min at 37 °C using FLUOstar Omega reader (BMG Labtech, Offenburg, Germany). Trolox solutions at various concentrations (0–250 µmol/L PBS) were used for calibration. The final ORAC values were calculated by using a regression equation between the Trolox concentration and the net area under the curve (AUC) as follows:

AUC = (1 + f1/f0 + f2/f0…fi/f0…)
(2)

where f0 is the initial fluorescence reading at time 0 min and fi is fluorescence reading at time i and expressed in µM of Trolox equivalents per gram of tested phytochemical (µM TE/g).

### 2.7. Statistical Analysis

Statistical analysis was performed using SigmaStat software. The differences in measured parameters between exposure and control groups were analysed for their statistical significance with the independent-samples two-sided *t* test. Significance was determined at the 5% level. Data are expressed as mean ± SD.

## 3. Results and Discussion

PM sample was collected in Kaunas, the second largest city of Lithuania. Winter was chosen for PM1 collection due to the steep increase in air pollution during heating season which is typical for Eastern Europe. Mass concentration of PM1 sample was 18 µg/m^3^. In comparison, average mass concentration of PM2.5 and PM10 collected in roadside and an urban area in Saitama (Japan) during winter season ranged from 28 to 43 µg/m^3^ [35]. BaP level in PM1 fraction was 3.08 ng/m^3^ strongly exceeding target value of 1 ng/m^3^ set by the European Commission. DMSO-extracted content of PM1 was employed in cell biology studies; recovery coefficients of the individual PAHs in the extract were 0.903–0.932. DMSO-extracted PAH mixture was diluted to the estimated BaP level in bronchial lining fluid of a person exposed to 3 ng/m^3^ BaP in the breathing air.

Detailed characterization of PAH content in PM1 was beyond the scope of this study, however our previous study on the same PM1 sample demonstrated that the concentrations of individual PAH constituents decreased in the following order (in ng/m^3^): benzo(a)pyrene (3.08 ± 1.37) > benzo(b)fluoranthene (2.71 ± 1.18) > chrysene (2.37 ± 1.06) > indeno(1,2,3-c,d)pyrene/dibenzo(a,h) anthracene (2.15 ± 0.96) > benzo(g,h,i)perylene (1.79 ± 0.79) and others [30]. The total PAH concentration in the tested sample was 17.75 ng/m^3^; for comparison, in some other European cities PAH concentration in winter season varied in a wide range: in Belgrade 50–100 ng/m^3^ [36], Oporto 16.3 ng/m^3^, Florence (7.75 ng/m^3^) and Athens (3.44 ng/m^3^) [37].

### 3.1. Protective Effects of Phytochemicals in PAH-Treated BEAS-2B Cells

**Figure 1:**
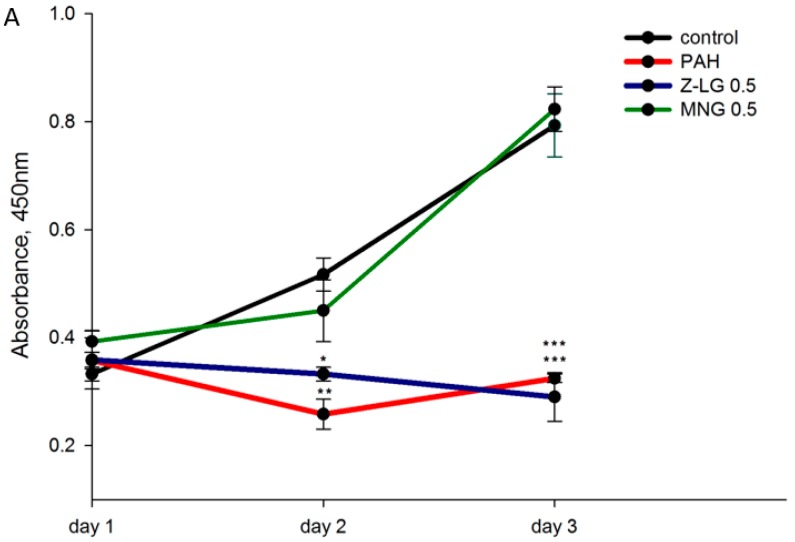

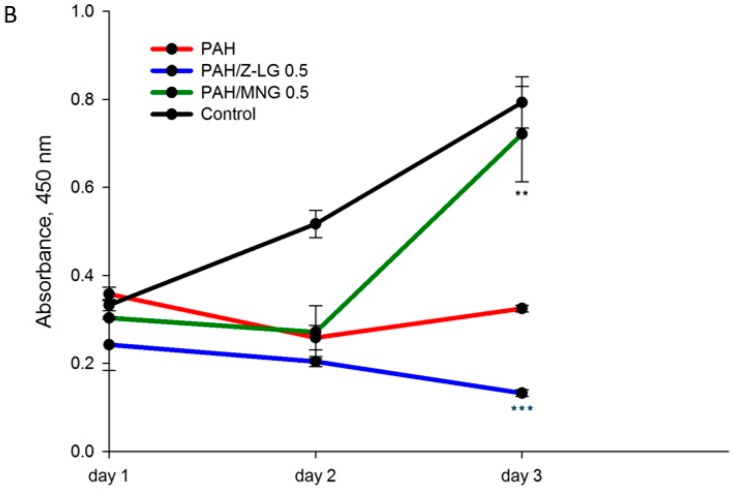
The effects of polycyclic aromatic hydrocarbons (PAH) extract (containing 2nM of benzo(a)pyrene (BaP)), Z-ligustillide (Z-LG, 0.5 µg/mL), mangiferin (MNG, 0.5 µg/mL) or combination thereof on proliferation of human bronchial epithelial cells. In the graph A a 3-day proliferation rate of bronchial epithelium BEAS-2B cells exposed to PAH extract (red plot) is represented in comparison to control (black plot), MNG- (green plot) and Z-LG exposed cells (blue plot). Proliferation rate of cells exposed to the combinations of PAH/MNG (green plot) and PAH/Z-LG (blue plot) is shown in the graph B and compared to the PAH-exposed cells (red plot). Proliferation was monitored by Cell Counting Kit-8 (CCK-8) assay measuring adsorption at 450 nm every 24 h. Data are presented as means ± SD. Each point represents at least three independent experiments. Significance is indicated as follows: * for *p* < 0.05, ** for *p* < 0.01, *** for *p* < 0.001.

Bronchial epithelium acts as a physicochemical barrier and plays a crucial role in initiating and augmenting defence mechanisms [38]. Therefore, bronchial epithelial cells (BEAS-2B) have been chosen as a model system to evaluate cytotoxicity induced by PM1-derived PAH mixture and possible protective effect of selected phytochemicals. So far as the exposure to toxicants may result in either partial damage of cellular function or cell death, time dependent cytotoxicity was evaluated. For this BEAS-2B cell cultures in confluent monolayers were exposed to PAH extract (containing 2 nM of BaP), *Z*-LG (0.5 µg/mL), MNG (0.5 µg/mL) or combination thereof for 24, 48 and 72 h and cell viability was measured by metabolic activity-based WST-8 (CCK-8) assay, where the amount of the formazan dye, generated by the activities of dehydrogenases in the cells, is directly proportional to the number of living cells. The results indicate a significant time-dependent decrease in PAH-exposed BEAS-2B cells viability, especially on day 3 (*p* < 0.01 for the day 2 and *p* < 0.001 for the day 3; Figure 1) in comparison to control cells. The decrease in cell viability observed after PAH exposure can be explained by an early toxicity which alters their proliferation [39]. Moreover, in agreement with our results, previous studies from Puerto Rico suggested that organic compounds in PM10 (from an urban/industrialized site) played a major role in the cytotoxicity observed in bronchial epithelial cells [40]. MNG-treated cells demonstrated viability comparable to the control cells, while Z-LG-treated cells exhibited significantly lower proliferation rate in comparison to the controls (*p* < 0.05 for the day 2 and *p* < 0.001 for the day 3), similarly to PAH-treated cells.

Importantly, cell proliferation was restored to the control level (*p* < 0.01 on the day 3) in MNG/PAH treated cells in comparison to PAH only exposed cells (green vs red plot in the Figure 1, lower panel). Treatment with Z-LG/PAH combination significantly reduced BEAS-2B proliferation rate in comparison to the control cells (*p* < 0.001; Figure 1, lower panel) and this reduction was even stronger than in PAH-treated cells. Similar protective action of MNG was also demonstrated in a previous study where pre-treatment of rat adrenal pheochromocytoma cells N2A with MNG resulted in an appreciable recovery of cell viability at 24 and 48 h, relative to cells dosed with cytotoxic 1-methyl-4-phenyl-pyridine ion [39]. Our data on the downregulation of proliferation in Z-LG-exposed cells is also in agreement with the previous report [41], where *Z*-LG treatment decreased viability of neuroblastoma cells PC12 in a dose-dependent manner and cells exposed to *Z*-LG/dopamine combination exhibited even stronger toxicity. Different effect was observed by Yu et al. [42], who reported that H_2_O_2_ significantly decreased the viability of PC12 cells, which was attenuated by *Z*-LG treatment in dose-dependent manner. Divergent effects reported on Z-LG suggest that its actions are highly target cell-, tissue- and, also, dose-specific. Some previous studies indicate that the main targets of *Z*-LG action are brain and vascular systems [43,44].

### 3.2. PAH Extract-Induced Oxidative Stress in Bronchial Epithelium and Effects of MNG and Z-LG

**Figure 2:**
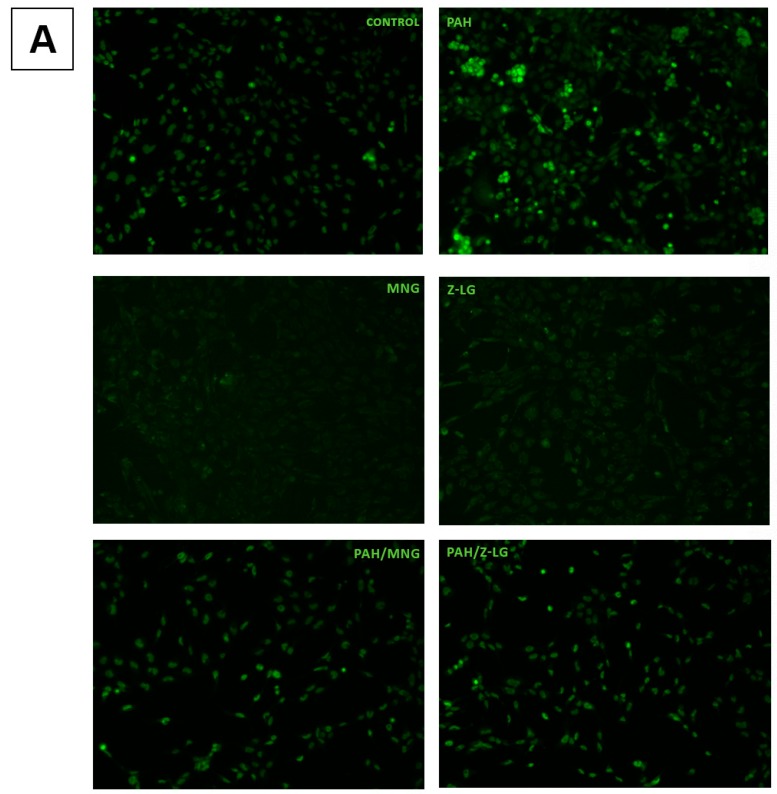

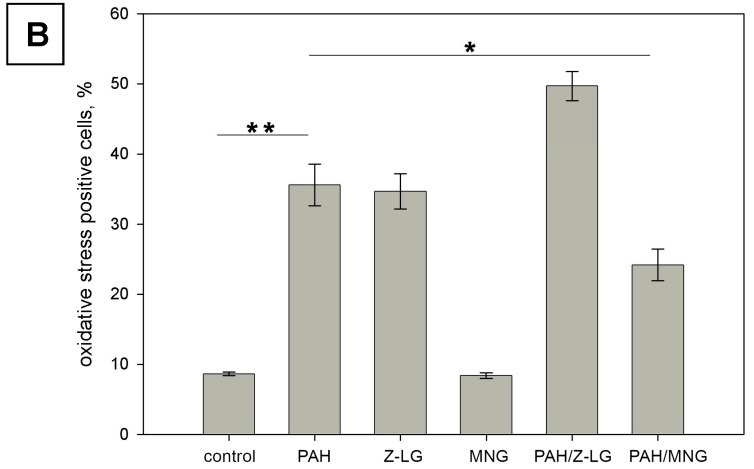
The effects of polycyclic aromatic hydrocarbons (PAH) extract (containing 2 nM of benzo(a)pyrene (BaP)), Z-ligustillide (Z-LG, 0.5 µg/mL), mangiferin (MNG, 0.5 µg/mL) or combination thereof on the levels of oxidative stress in BEAS-2B cells. (**A**) PAH-induced oxidative stress in BEA-2B cells was assessed by means CellROX^®^ Green Reagent. Green areas represent cells with ongoing oxidative stress. Magnification 20X. (**B**) Oxidative stress undergoing cell were identified using Muse^®^ Oxidative Stress Kit and Muse^®^ cytometer. Data are presented as mean ± SD from 3 independent experiments. Significance is indicated as follows: * for *p* < 0.05, ** for *p* < 0.01.

Previous studies using in vitro and in vivo models suggest that PAH generate reactive oxygen species (ROS), resulting in oxidative stress and inflammatory responses, which account for the prevalence and exacerbation of respiratory diseases [45]. Since oxidative stress is one of the major mechanisms by which PM exert adverse biological effects [46], we have assessed oxidative status in PAH-exposed BEAS-2B cells with or without MNG and *Z*-LG treatment. BEAS-2B cells were loaded with CellROX^®^ green reagent to observe ROS generation and oxidative stress induction in live cells. Representative micrographs of fluorescent cells are presented in Figure 2A. These results were further confirmed and quantitated in the separate experiment, by flow cytometry (Figure 2B).

As shown in Figure 2, PAH-exposed cells exhibit higher level of organized green fluorescence (indicative of intracellular ROS levels) in comparison to the control cells (visualized in Figure 2A, quantified in Figure 2B, *p* < 0.01). Next, potential protective effect of MNG (0.5 µg/mL) and Z-LG (0.5 µg/mL) were tested treating BEAS-2B cells with phytochemicals alone or in combinations with PAH. Results obtained underline the different effect of tested constituents. MNG and Z-LG treatment did not affect oxidative stress levels in BEAS-2B (Figure 2). However, when PAH and MNG were added together, the levels of oxidative stress were significantly reduced in comparison to PAH-exposed cells (*p* < 0.05). Oppositely, combined Z-LG/PAH treatment induced even more oxidative stress that PAH treatment alone. MNG action could be related to high antioxidant activity reported in previous studies, for example, induced intracellular ROS levels in normal kidney epithelial cells were significantly downregulated by MNG treatment. The mechanism behind this effect might be MNG ability to restore the levels of cellular metabolites and phase II antioxidant enzymes that are markedly decreased in oxidative stress [47]. It was also recently reported that aqueous extract of *Davallia mariesii* containing epicatechin and MNG as its major active compounds attenuates 6-hydroxydopamine-induced oxidative damage and apoptosis in B35 cells through inhibition of caspase cascade and activation of PI3K/AKT/GSK-3 pathway [48].

Although both phytochemical compounds usually are characterized as antioxidant materials, in this study *Z*-LG increased level of ROS in the BEAS-2B cells similarly to PAH extract. Moreover, *Z*-LG also exhibited an additive negative effect in cells exposed to PAH/Z-LG combination in comparison to PAH-treated epithelium. This correlates with the results from cytotoxicity assay, where *Z*-LG has displayed additive effect to PAH-induced cytotoxicity as well. Contrasting effects of *Z*-LG on intracellular redox status were observed in previous studies. Studies undertaken by Qi et al. [41] have also demonstrated that *Z*-LG stimulates ROS formation in a concentration-dependent manner in PC12 cells. Contrary results were obtained in other studies [44], where treatment with *Z*-LG has significantly suppressed ROS production in vascular smooth muscle cells. The controversy around efficacy and targets of antioxidants is based on many various factors, like dose, cell type, method of delivery, time and duration of exposure [49].

### 3.3. Effects of MNG on Epithelial Wound Healing

The airway epithelium is continuously subjected to various detrimental factors including environmental pollutants, airborne pathogens, tobacco smoke and so forth. In order to sustain normal epithelial structure and function after injuries airway epithelium relies on several intrinsic mechanisms including migration, proliferation and differentiation to maintain barrier integrity [50,51]. Motility changes in various air-pollution exposed cells are well described [30,52].

**Figure 3:**
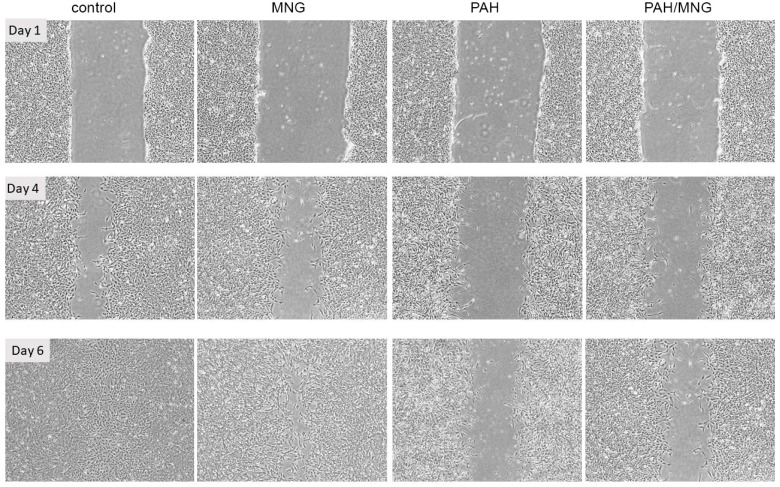
The effects of polycyclic aromatic hydrocarbons (PAH) extract (containing 2 nM of benzo(a)pyrene (BaP)), mangiferin (MNG, 5 µg/mL) or combination thereof on human bronchial epithelial cell wound healing properties. Representative images of wound healing assay in the monolayers of bronchial epithelium under influence of PAH extract, mangiferin and combination of those two compared to the controls. Pictures were taken at day 1, 4 and 6 of the exposure.

To investigate the wound healing properties of epithelial cells, we have used an in vitro model of epithelial injury, which involves generating a linear scratch in confluent monolayers of BEAS-2B cells. We have found that exposure to PAH (containing 2 nM of BaP) inhibits cells’ ability to cover scratched area when compared to control (solvent only exposed) cells (Figure 3). This effect could be related to the downregulated level of barrier proteins such as e-cadherin and β-catenin [30]. Addition of MNG (0.5 µg/mL) to the cell culture medium produced an ameliorating effect on PAH-induced decrease in wound healing capacity. Within 6 days wounds were complete covered in control and MNG treated cultures, while scratch was still wide open in PAH-exposed cultures and visible in PAH/MNG combination exposed ones. The positive effect of MNG on cell motility was also observed by other researchers [53], who reported that MNG increased bovine aortic endothelial cells migration without an increase in cell proliferation. Previous studies demonstrated that pre-treatment with MNG may revert ischemia/reperfusion-suppressed β-catenin content, partly through inhibiting glycogen synthase kinase-3β (GSK-3B), nuclear factor-kappaB (NF-κB) and oxidative stress [54].

### 3.4. In Vitro Antioxidant Activity

Considering divergent effects of MNG and Z-LG on PAH-exposed bronchial epithelial cells observed in our study antioxidant properties of these phytochemicals was tested using several widely used chemical in vitro assays, namely ABTS^•+^ and DPPH^•^ scavenging capacity, ferric reducing antioxidant power (FRAP) and oxygen radical absorbance capacity (ORAC). The results are expressed as Trolox equivalent antioxidant capacity (TEAC) in µmol TE/g.

MNG demonstrated remarkably higher antioxidant activity than *Z*-LG (Table 1). For instance, MNG was about 93 times stronger DPPH^•^ scavenger than *Z*-LG, while ABTS^•+^ scavenging capacity of MNG was 37 time higher than that of *Z*-LG. Previous studies on the radical scavenging activity toward DPPH^•^ indicated that the concentration of *Z*-LG needed to decrease the initial DPPH^•^ concentration by 50% (IC_50_) was 268.3 µg/mL, while IC_50_ of trolox was 2.6 µg/mL, that is, 100 times lower [22]. Another study [55] reported IC_50_ values for MNG and rutin 5.80 and 5.56 µg/mL, respectively. The mechanism of ABTS^•+^ and DPPH^•^ scavenging by the antioxidant is based on a single electron transfer [56]. MNG was also very efficient antioxidant in FRAP assay, which is based on electron-transfer reaction as well [57]; whereas *Z*-LG was weaker phytochemical in this assay more than 11 times.

The ORAC method evaluates chain breaking antioxidant capacity against peroxyl radicals via hydrogen atom transfer and is more relevant to the biological systems [58,59]. In this assay MNG and *Z*-LG demonstrated ORAC values of 13,675 and 1166 µmol TE/g, respectively. For comparison, ORAC values reported for other well-known antioxidant compounds such as caffeic acid, quercetin, gallic acid were 1528, 1335 and 6970 µmol/mg, respectively [60]. It should be noted that in ORAC assay MNG was remarkably stronger antioxidant than Trolox (3.4 g TE/g), which is in agreement with the previously reported data [48].

These results correlate well with the results obtained in cell biology assays, where MNG significantly decreased oxidative stress induced by PAH. The number of hydroxyl groups and catechol moiety in the MNG molecule could be correlated to its high antioxidant capacity [55]. The divergent effect of *Z*-LG on cells oxidative state might be related to the different solubility of these compounds (MNG is hydrophilic, while Z-LG lipophilic) and/or explained by different interaction mechanism with the target cells, which were not interfering in acellular systems. This may be also related to divergent bioavailability of those two compounds in the airway epithelium. Previous studies have reported that regardless of having the highest total phenolic, total flavonoids content and ORAC values, phytochemicals rich Shanlihong variety of *C. pinnatifida* did not rank first for cellular antioxidant quality. It suggested that some compounds present in the extracts were not so effective in cell cultures due to poor adsorption or poor association with the target cell membrane [61].

## 4. Conclusions

In the present study we have focused on the cytotoxic, oxidative and wound healing changes induced in bronchial epithelial cells (BEAS-2B) by urban fine PM–derived PAH mix alone or in combination with well-known phytochemicals MNG and Z-LG. The study has revealed that exposure to PAH (containing 2 nM of BaP) significantly decreases bronchial epithelial cell proliferation capacity, induces oxidative stress and inhibits wound healing properties. Cell proliferation assay has shown, that MNG (0.5 µg/mL) treatment may reverse PAH induced cytotoxicity, while Z-LG (0.5 µg/mL), alone or in combination with PAH, downregulates proliferation. Although both tested phytochemicals possessed in vitro antioxidant activity, their cellular effects were divergent. Surprisingly, Z-LG induced oxidative stress in the treated cells, rather than protecting from it. Oppositely, MNG significantly inhibited ROS production and restored proliferation and wound healing capabilities in the exposed cells. Our findings indicate that exposure of airway epithelium to urban air pollution leads to acute adverse effects that can be modulated by MNG.

## Figures and Tables

**Table 1:** Antioxidant capacity characteristics of MNG and *Z*-LG.

Sample	DPPH, µM TE/g	ABTS, µM TE/g	FRAP, µM TE/g	ORAC, µM TE/g
MNG	4282 ± 246 ^b^	7227 ± 226 ^b^	6750 ± 2.13 ^b^	13,675 ± 540 ^b^
*Z*-LG	45.93 ± 2.44 ^a^	194.3 ± 5.96 ^a^	597.8 ± 33.9 ^a^	1166 ± 47.4 ^a^

Data are presented as mean ± SD from 3 independent experiments. Columns with different letters differ significantly at *p* < 0.0001. DPPH, diphenyl-1-picryhydrazyl; ABTS, 2,2-azino-bis(3-ethylbenzothiazoline-6-sulfonic acid); FRAP, ferric reducing antioxidant power; ORAC, oxygen radical absorbance capacity.
